# Supplementary figures and images for: Daptomycin for Treatment of S. Epidermidis Endocarditis in an Extremely Preterm Neonate—Outcome and Perspectives
Source: Children (Basel). 2022 Mar 24;9(4):457. doi: 10.3390/children9040457 (PMC9030184; doi:10.3390/children9040457)

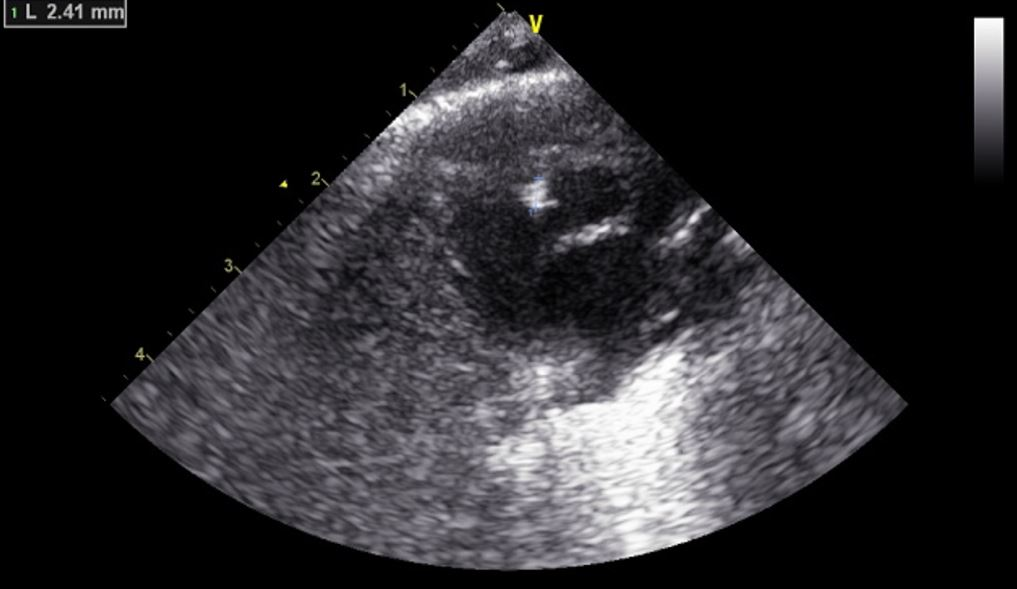

Supplement: Supplementary file 1 [file children-09-00457-s001.zip › Echocardiography 1.tiff]

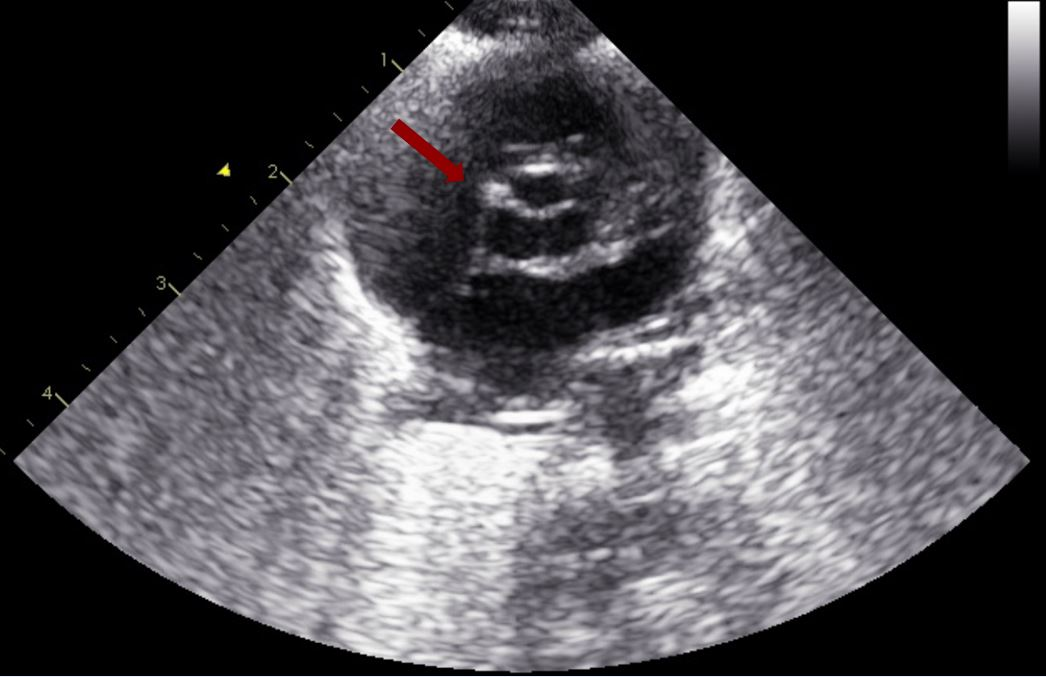

Supplement: Supplementary file 1 [file children-09-00457-s001.zip › Echocardiograpy.tiff]
